# Variation in the effects of family background and birth region on adult obesity: results of a prospective cohort study of a Great Depression-era American cohort

**DOI:** 10.1186/s12889-015-1870-7

**Published:** 2015-06-05

**Authors:** Hui Zheng, Dmitry Tumin

**Affiliations:** Department of Sociology, The Ohio State University, Columbus, OH 43210 USA

**Keywords:** Obesity, Early-life conditions, Mother’s education, Birth place, Gender, Life course

## Abstract

**Background:**

Studies have identified prenatal and early childhood conditions as important contributors to weight status in later life. To date, however, few studies have considered how weight status in adulthood is shaped by regional variation in early-life conditions, rather than the characteristics of the individual or their family. Furthermore, gender and life course differences in the salience of early life conditions to weight status remain unclear. This study investigates whether the effect of family background and birth region on adult obesity status varies by gender and over the life course.

**Methods:**

We used data from a population-based cohort of 6,453 adults from the Health and Retirement Study, 1992–2008. Early life conditions were measured retrospectively at and after the baseline. Obesity was calculated from self-reported height and weight. Logistic models were used to estimate the net effects of family background and birth region on adulthood obesity risk after adjusting for socioeconomic factors and health behaviors measured in adulthood. Four economic and demographic data sets were used to further test the birthplace effect.

**Results:**

At ages 50–61, mother’s education and birth region were associated with women’s obesity risk, but not men’s. Each year’s increase in mother’s education significantly reduces the odds of being obese by 6 % (OR = 0.94; 95 % CI: 0.92, 0.97) among women, and this pattern persisted at ages 66–77. Women born in the Mountain region were least likely to be obese in late-middle age and late-life. Measures of per capita income and infant mortality rate in the birth region were also associated with the odds of obesity among women.

**Conclusions:**

Women’s obesity status in adulthood is influenced by early childhood conditions, including regional conditions, while adulthood health risk factors may be more important for men’s obesity risk. Biological and social mechanisms may account for the gender difference.

**Electronic supplementary material:**

The online version of this article (doi:10.1186/s12889-015-1870-7) contains supplementary material, which is available to authorized users.

## Background

Recent epidemiological studies of obesity in adults have identified prenatal and early childhood conditions as important contributors to weight status in later life [1–9]. These studies show that obesity status in adulthood is rooted in childhood conditions, supporting the latency or critical period model [10]. The critical period model emphasizes that exposures acting during a specific period have long-lasting effects on anatomical structure or physiological function, and that these effects are not substantially altered by later experience. This model describes a process of “biological programming”, and appears in Barker and colleagues’ exposition of the “fetal origins of adult disease” [11–14]. The effect of early-life conditions on adulthood obesity indicates that prevention of adulthood obesity should begin in early life. But, to date, few studies have considered how weight status in adulthood is shaped by regional variation in early-life conditions, rather than characteristics of the individual or family. Birth region can have long-lasting effects on health status, disease burden, and mortality [15–17]. Birth region may be even more influential for adulthood health than the region of current residence, making it a potentially important source of variation in obesity status within cohorts reaching middle age and beyond [18]. In this study, we test for an association between birth region and adult obesity.

The association between birth region and adult obesity is likely gendered, as gender differences in the effects of other early-life conditions on the risk of adult obesity have been reported in several studies. One study found that early-life conditions matter only among men [19], but other studies have argued that early-life conditions are salient only for women [3, 5]. The effect of early-life conditions on obesity in adulthood for women, but not men, was related to several possible explanations. First, men are more likely than women to experience upward social mobility, attenuating the negative impact of early childhood disadvantages [6], whereas women are more likely to experience discrimination in education and work throughout their life course, exacerbating early life disadvantages [20]. Second, due to social pressure for women to be slim, especially among women of higher socioeconomic status (SES), women from advantaged backgrounds would be more likely to maintain low body weight than women born in families with lower status [7]. Consistent with this argument, women from higher SES neighborhoods are more likely to have a healthy diet and consume fewer high-fat foods than women from lower-SES neighborhoods, a pattern that was not observed among men [21]. Third, obesity is more stigmatized among women than men, which may limit upward mobility for obese women more so than obese men [22], exacerbating the effect of early life disadvantages on adult obesity among women.

The above three explanations emphasize socially constructed differences in gender roles, norms of physical appearance, and resources in the family of origin that shape the gender-patterned consequences of early life conditions for obesity in adulthood. A biological explanation, however, may highlight a gender difference in “the body’s encoding and storage of early harmful exposures” [23]. Some studies find a stronger impact of childhood socioeconomic conditions among women than men on metabolic syndrome, low HDL cholesterol levels and dyslipidemia [24–26]. These studies, however, cannot tell if biological or social processes that unfold over the life course are responsible for gender differences in the effect of early life conditions on the biomarker outcomes. In this study, our focus on birth region means that we examine a “neutral” early life condition that is not determined by family resources or socioeconomic status. If regional variation shapes obesity outcomes only among women, then this strengthens the case for biological mechanisms underlying observed gender differences in the effects of early-life conditions.

The effects of early-life conditions are known to evolve over the life course, and this pattern may also become apparent in the effects of birth region on obesity in later life. For example, as people age, the effect of early-life conditions on obesity risk might evolve from a direct effect (via physiological adaptation) to an indirect accumulation of behavioral risk factors [10]. Therefore, it is important to estimate the effect of birth region net of socioeconomic characteristics and health risk factors in adulthood to capture any independent and long-lasting influences of birth region on obesity. Furthermore, it is important to ensure that such region effects are not confounded by individual or family characteristics observed in early life. There may be socioeconomic differences across regions, meaning that an unadjusted correlation between birth region and later-life obesity would include both the effects of living in an advantaged (or disadvantaged) region and being raised in an advantaged (or disadvantaged) family.

This study explores the significance of birth region variation for obesity risk in adulthood in a Great Depression-era American cohort. This cohort was born in a period of pronounced regional differences in economic development and productivity [27, 28]. Although a full review of Depression-era regional contrasts is beyond the scope of this study, prior research has identified several important economic characteristics that varied across regions during this period. Specifically, crop failures and the concentration of the workforce in manufacturing were identified as important contributors to local effects of the Depression; whereas unemployment, income loss, and increasing infant mortality were identified as important consequences of the Depression that varied across regions [28]. We hypothesize that such regional differences—and other, unobserved differences between regions—have left a lasting imprint on weight trajectories of members of this cohort, and, particularly, have created birth-region inequalities in the odds of obesity that would persist to mid-life and beyond. In studying such regional variation in the odds of later-life obesity, we improve upon previous studies by using a large, nationally representative study with several waves at which body mass index (BMI) data are available; and controlling for a wide range of adulthood socioeconomic, behavioral and health characteristics. Taking advantage of these strengths, our study estimates the net effect of early life conditions, including birth region and family background, on obesity risk in late middle age; and tests whether this effect endures through old age and whether the effect differs by gender.

## Methods

### Data and participants

We used public data from the Health and Retirement Study (HRS), a nationally representative survey of Americans born between 1931 and 1941. HRS respondents and their spouses were initially interviewed in 1992, and re-interviewed at two-year intervals since then. Our primary data came from the 1992, 1998 and 2008 HRS waves. We restricted the analysis to 10,142 age-eligible (50-61 years old) respondents in the original 1992 HRS sample. Respondents were excluded if they were missing data on baseline covariates (2,013 cases), particularly birth region (9 % of age-eligible respondents missing data, including some born abroad), maternal education (9 % missing data), race/ethnicity (2 % missing data), and income or wealth (1 % missing data). Respondents were also excluded if they exited the study before 1998 or were missing data on retrospective measures of early-life socioeconomic status collected in that year (1,611 cases), and if they were underweight according to body mass index in 1992 (65 cases). These exclusions produced an analytic sample of 6,453 respondents who were 50–61 years old in 1992, and contributed retrospective data on their early-life socioeconomic status in the 1998 wave. Clearly, the retrospective data collection of early-life socioeconomic status in 1998, rather than at the 1992 baseline, caused almost half of the exclusions of original age-eligible respondents from our analytic sample (1,611 of 3,689 excluded respondents). As of the 2008 wave, 975 of the 6,453 included respondents had died and 687 were lost to follow-up. After excluding 56 respondents who became underweight according to body mass index by 2008, we reached a subsample of 4,735 respondents who contributed data on their body weight in 2008. We used this subsample to examine the effects of early-life conditions on obesity in old age (ages 66–77).

To explore how differences across birth regions may influence later-life obesity, we used several other data sets to assess region-year levels of regional characteristics that were related to the Great Depression. These characteristics included per capita income, employment, crop yield and infant mortality [28]. We used data from the U.S. Department of Commerce, Bureau of Economic Analysis to create a measure of per capita income in each region, in dollars per thousand people. We assessed regional employment using manufacturing employment and nonmanufacturing employment indices developed by Wallis [29]. We used data from the U.S. Department of Agriculture, National Agricultural Statistics Service to obtain measures of yield for four crops: wheat, corn and oats (in bushels per acre), and hay (in tons per acre). Finally, we included a measure of infant mortality rate using data from the U.S. Census Bureau.

### Measures

The primary outcome variables were obesity in middle and old age. Obesity was assessed using body mass index (BMI), defined as the ratio of weight in kilograms to the square of height in meters. Both height and weight were self-reported at the baseline 1992 interview, and weight was also self-reported in each subsequent interview. The obese group (BMI of at least 30 kg/m^2^) was compared to normal weight (BMI of 18.5–24.9 kg/m^2^) and overweight (BMI of 25–29.9 kg/m^2^) groups, following recent research that showed obesity, but not overweight, was associated with increased mortality risk among the elderly [30]. Respondents in the underweight (BMI lower than 18.5 kg/m^2^) group were not analyzed due to the association between underweight and the wasting effects of disease among elderly adults [31].

The first set of independent variables comprised early life demographic and socioeconomic factors: birth date (in century-month code), race/ethnicity (non-Hispanic White, non-Hispanic Black, or Hispanic), birth region (New England, Middle Atlantic, East North Central, West North Central, South Atlantic, East South Central, West South Central, Mountain, and Pacific), mother’s years of schooling, and self-reported financial status from birth to age 16 (“pretty well off financially,” “about average,” and the reference group, “poor”). We also included binary measures of the respondent’s family having moved because of financial difficulties; the respondent’s family having received help from relatives because of financial difficulties; and the respondent’s father having lost his job while the respondent was young.

Next, we included mid-life social and economic variables assessed at the 1992 wave: marital status (never married, married, separated, divorced, widowed, or living with a partner), years of schooling, income, net wealth, and region of current residence (Midwest, South, West, and Northeast, which is the reference group). Four rather than nine regional categories were used here to reduce multicollinearity problems. We also included a measure of the respondent’s lifetime occupation, defined as the occupational category describing the respondent’s longest-held job. Occupations were divided into white-collar (managers, professionals, salesmen); pink-collar (clerical and service industry workers); blue-collar (farmers, operators, craftsmen); and having never worked (the reference group). Finally, we included measures of the respondent’s health behaviors at midlife that could have contributed to obesity status: smoking status (never-smoker, former smoker, or current smoker); and a binary measure of engaging in vigorous physical activity at least three times per week.

### Statistical analysis

We fitted logistic models of obesity (with normal weight and overweight combined as the reference group) in 1992 for men and women separately. In the first model, BMI status in 1992 was a function of demographic factors and early life conditions only. In the second model, BMI status in 1992 was predicted by demographic factors, early life conditions, and mid-life socioeconomic status and health behaviors. We then examined how these associations change later in the life course by fitting the same logistic models to obesity (with normal weight and overweight as the reference group) in 2008, calculated using self-reported weight in 2008 and self-reported height from the baseline (1992) interview. Then, we tested which regional measures, including income, employment, crop yield, and infant mortality rate were correlated with obesity in mid- and later life net of individual-level covariates. All analyses were performed using Stata/MP 13.1 (College Station, TX: StataCorp).

## Results

Table 1 describes the analytic sample. As of 1992, 26 % of women and 21 % of men were obese. Among respondents who also contributed data on BMI in 2008, obesity became more common, with 31 % of both men and women being obese at ages 66–77. Table 2 shows results from logistic regression models predicting obesity in 1992. (A full table of coefficients, is presented in online Additional file 1: Table S1). After adjusting for demographic factors, each year’s increase in mother’s education significantly reduces the odds of being obese by 6 % among women. Individual early life economic conditions (having been pretty well off or about average financially rather than poor; father’s job loss; receipt of financial help from relatives; or having moved for financial reasons) did not predict women’s odds of being obese in late-middle age.

**Table 1:** Characteristics of participants in the Health and Retirement Study, United States, 1992

	Total (*n* = 6,453)		Women (*n* = 3,539)		Men (*n* = 2,914)	
	Median (IQR)	%	Median (IQR)	%	Median (IQR)	%
Normal weight^a^		35.7		41.1		29.1
Overweight^b^		40.7		33.4		49.5
Obesity^c^		23.7		25.5		21.4
Demographics						
Birth date (months since Jan. 1900)	446 (411–478)		447 (412–480)		443 (410–475)	
Non-Hispanic white		80.1		78.4		82.1
Non-Hispanic black		15.6		17.5		13.4
Hispanic		4.3		4.2		4.5
Early life factors						
Mother’s years of schooling	10 (8–12)		9 (8–12)		10 (8–12)	
Pretty well off financially		5.3		5.3		5.1
About average financially		63.9		65.1		62.6
Poor		30.8		29.6		32.3
Father lost job		18.3		17.6		19.0
Financial help from relatives		11.6		11.0		12.3
Moved for financial reasons		16.8		15.6		18.1
Born in New England		4.3		3.8		4.9
Born in Middle Atlantic Division		14.9		15.2		14.5
Born in East North Central Division		18.6		18.0		19.3
Born in West North Central Division		11.6		11.2		12.0
Born in South Atlantic Division		19.2		19.6		18.6
Born in East South Central Division		11.1		11.4		10.8
Born in West South Central Division		11.4		12.0		10.7
Born in Mountain Division		4.0		3.9		4.1
Born in Pacific Division		5.1		5.1		5.0
Late-middle life socio-demographic factors						
Years of schooling	12 (12–14)		12 (12–14)		12 (12–15)	
Income (1992 dollars)	40,000 (21,800–63,592)		35,200 (19,000–59,284)		46,000 (26,804–69,500)	
Net wealth (1992 dollars)	105,000 (38,700–241,000)		99,600 (34,000–235,000)		111,500 (43,200–249,000)	
White-collar lifetime occupation		38.1		34.6		42.3
Pink-collar lifetime occupation		27.2		41.3		10.1
Blue-collar lifetime occupation		27.3		12.0		45.9
Never worked		7.4		12.1		1.7
Married		76.2		70.8		82.7
Partner		2.2		1.5		3.0
Separated		2.3		2.6		2.0
Widowed		5.6		8.9		1.4
Never married		3.4		3.5		3.2
Divorced		10.4		12.7		7.6
Live in Northeast Region		16.0		16.7		15.1
Live in Midwest Region		27.9		27.9		27.8
Live in South Region		40.7		40.1		41.4
Live in West Region		15.5		15.3		15.8
Late-middle life behavior-health factors						
Current smoker		26.2		24.9		27.7
Former smoker		36.6		29.3		45.5
Never smoker		37.2		45.8		26.8
Vigorous physical activity (> = 3 times per week)		22.4		22.7		22.2

Abbreviations: *BMI* body mass index, *IQR*, interquartile range

BMI categories: ^a^normal weight, BMI between 18.5 and 24.9 kg/m^2^; ^b^overweight, BMI between 25 and 29.9 kg/m^2^; ^c^obesity, BMI equal to or greater than 30 kg/m^2^

**Table 2:** Life course factors associated with obesity: odds ratios from logistic models at age 50-61

Covariate	Women (*n* = 3,539)				Men (*n =* 2,914)			
	Adjusted for demographic factors^a^		Adjusted for late-middle life socio-demographic and behavior-health factors^b^		Adjusted for demographic factors^a^		Adjusted for late-middle life socio-demographic and behavior-health factors^b^	
	OR	95 % CI	OR	95 % CI	OR	95 % CI	OR	95 % CI
Mother’s years of schooling	0.94	0.92, 0.97	0.96	0.93, 0.99	0.99	0.96, 1.02	1.00	0.97, 1.03
Pretty well off financially	1.10	0.75, 1.63	1.38	0.92, 2.08	0.64	0.40, 1.04	0.73	0.45, 1.19
About average financially	1.00	0.83, 1.21	1.04	0.86, 1.26	0.84	0.68, 1.03	0.83	0.68, 1.03
Father lost job	0.94	0.75, 1.17	0.92	0.74, 1.15	1.20	0.95, 1.52	1.21	0.95, 1.54
Received financial help from relatives	1.16	0.89, 1.51	1.21	0.92, 1.58	0.83	0.61, 1.12	0.85	0.63, 1.15
Family moved for financial reasons	1.00	0.79, 1.27	0.96	0.75, 1.22	0.79	0.61, 1.03	0.80	0.61, 1.04
Born in New England Division	1.74	1.14, 2.68	1.42	0.86, 2.35	1.46	0.93, 2.28	1.66	0.99, 2.79
Born in Middle Atlantic Division	1.43	1.08, 1.88	1.15	0.81, 1.65	1.22	0.88, 1.69	1.34	0.90, 2.00
Born in East North Central Division	1.35	1.03, 1.76	1.03	0.73, 1.45	1.35	1.00, 1.83	1.37	0.94, 1.98
Born in West North Central Division	1.07	0.78, 1.47	0.80	0.54, 1.19	1.15	0.81, 1.64	1.12	0.73, 1.72
Born in East South Central Division	0.84	0.63, 1.12	0.77	0.57, 1.04	1.08	0.77, 1.52	1.05	0.74, 1.49
Born in West South Central Division	0.96	0.72, 1.28	0.92	0.68, 1.25	1.12	0.78, 1.59	1.09	0.76, 1.58
Born in Mountain Division	0.65	0.39, 1.11	0.64	0.35, 1.17	0.90	0.52, 1.56	0.94	0.51, 1.73
Born in Pacific Division	0.83	0.53, 1.29	0.88	0.52, 1.49	1.07	0.66, 1.72	1.13	0.64, 2.00

Abbreviations: *CI* confidence interval, *OR* odds ratio

^a^Adjusted for birth date, and race-ethnicity

^b^Adjusted for birth date, race-ethnicity, marital status, education, income, net wealth, lifetime occupation, current residential place, smoking status, and physical activity

Women who were born in the New England, Middle Atlantic, and East North Central divisions were significantly more likely to be obese at midlife than women born in the South Atlantic division. Women born in New England had the highest odds of obesity, whereas women born in the Mountain division had the lowest odds of obesity at midlife. After adjusting for late-middle life socio-demographic and health factors, the effects of mother’s education (OR = 0.96, 95 % CI: 0.93, 0.99) remained significant and in the same direction as in the previous model. In the adjusted model, some inter-region differences in obesity were attenuated, although women born in New England remained significantly less likely to be obese at midlife than women born in the Mountain Division (OR = 1.46/0.64 = 2.21; 95 % CI = 1.08, 4.50). Among men, all early life factors, including mother’s education and birth region, had no significant effect on obesity in late-middle adulthood. We tested the significance of these gender differences by fitting a model to the combined sample of men and women, and adding terms for gender interactions with mother’s education and birth region. The effect of mother’s education was significantly stronger among women than men.

Table 3 shows results from logistic regression models for women and men who were 66–77 years old as of the 2008 HRS wave. (A full table is included in the online Additional file 1: Table S2). At these ages, mother’s education maintains a significant effect on women’s obesity risk. Moreover, the size of this effect does not decline from ages 50–61 to ages 66–77. There also remain significant differences in late-life obesity across birth region, with or without adjustment for individual characteristics at midlife. Particularly, women born in the East South Central and Mountain divisions are significantly less likely to be obese in late life than women born in the South Atlantic division. Among men, all early life factors have no effect on obesity risk in late age, as in the results for obesity status at ages 50–61.

**Table 3:** Life course factors associated with obesity: odds ratios from logistic models at age 66–77

Covariate	Women (*n* = 2,672)				Men (*n* = 2,063)			
	Adjusted for demographic factors^a^		Adjusted for late-middle life socio-demographic and behavior-health factors^b^		Adjusted for demographic factors^a^		Adjusted for late-middle life socio-demographic and behavior-health factors^b^	
	OR	95 % CI	OR	95 % CI	OR	95 % CI	OR	95 % CI
Mother’s years of schooling	0.95	0.92, 0.98	0.96	0.94, 0.99	0.99	0.96, 1.03	1.01	0.98, 1.05
Pretty well off financially	0.93	0.61, 1.43	1.11	0.72, 1.72	0.71	0.42, 1.17	0.79	0.47, 1.33
About average financially	1.05	0.85, 1.29	1.08	0.88, 1.33	0.99	0.79, 1.23	1.00	0.80, 1.25
Father lost job	0.93	0.74, 1.17	0.90	0.71, 1.14	1.11	0.87, 1.43	1.08	0.84, 1.40
Received financial help from relatives	1.10	0.83, 1.45	1.12	0.85, 1.49	1.18	0.87, 1.60	1.20	0.88, 1.63
Family moved for financial reasons	1.04	0.81, 1.34	1.02	0.79, 1.32	0.92	0.70, 1.20	0.95	0.72, 1.24
Born in New England Division	1.07	0.66, 1.73	1.06	0.61, 1.85	0.83	0.50, 1.38	0.75	0.42, 1.35
Born in Middle Atlantic Division	1.15	0.85, 1.55	1.13	0.77, 1.66	1.01	0.71, 1.43	0.91	0.59, 1.39
Born in East North Central Division	1.03	0.77, 1.38	0.93	0.65, 1.34	1.30	0.95, 1.77	1.16	0.79, 1.70
Born in West North Central Division	1.07	0.77, 1.48	0.95	0.64, 1.41	1.07	0.75, 1.53	0.91	0.58, 1.41
Born in East South Central Division	0.71	0.52, 0.98	0.68	0.49, 0.94	0.90	0.62, 1.31	0.87	0.59, 1.27
Born in West South Central Division	1.05	0.77, 1.43	1.02	0.74, 1.40	0.88	0.60, 1.29	0.86	0.58, 1.27
Born in Mountain Division	0.40	0.22, 0.73	0.43	0.22, 0.84	0.88	0.51, 1.52	0.81	0.44, 1.50
Born in Pacific Division	1.29	0.85, 1.95	1.35	0.81, 2.23	0.94	0.57, 1.56	0.87	0.48, 1.59

Abbreviations: *CI* confidence interval, *OR* odds ratio

^a^Adjusted for birth date, and race-ethnicity

^b^Adjusted for birth date, race-ethnicity, marital status, education, income, net wealth, lifetime occupation, current residential place, smoking status, and physical activity

### Regional factors associated with obesity among women aged 50–61

In Table 4, we explore whether specific regional-level factors are associated with women’s odds of being obese in late-middle age. The logistic regression models shown in Table 4 include all individual-level covariates from Table 2, and one or more additional terms for regional factors, which include measures of crop yield, per capita income, employment, and infant mortality. In Models 1–4, regional factors are entered separately from one another; in Model 5, regional factors that were significant on their own are entered jointly; and in Model 6, all regional factors are entered simultaneously. Odds ratios from individual-level covariates are not shown in the table. Birth region dummies were not included due to collinearity with specific regional measures. Every 1,000 unit increase in per capita income (dollars per 1,000 people) was associated with a significant increase in the odds of obesity by about 83 %, and a higher regional infant mortality rate was significantly associated with reduced obesity risk in late-middle adulthood among women. When entered simultaneously into Model 5, per-capita income remained positively associated with women’s odds of obesity (OR = 2.37; 95 % CI: 1.01, 5.52) whereas infant mortality rate was no longer significantly associated with this outcome. In Model 6, all regional covariates were entered regardless of their significance in Models 1–4. A likelihood ratio test comparing Model 6 to Model 5 showed that adding the crop yield and employment variables (which were not independently related to women’s obesity risk) failed to improve model fit (p = 0.401) over the more parsimonious Model 5. Fitting Models 1–6 to the men’s subsample revealed no significant associations between specific characteristics of the birth region and men’s odds of obesity in later life (Additional file 1: Table S3). Table 4 suggests that per capita income and infant mortality in the birth region may contribute to women’s obesity risk in adulthood net of individual characteristics. Additional file 1: Table S4 shows that the New England, Middle Atlantic, East North Central, and Pacific divisions had higher per capita income and lower infant mortality rates compared to other divisions. We examined whether the results in Table 4 reflected the exceptionally high obesity rate among women born in New England. Excluding these women from Models 1–4 in Table 4, we found that infant mortality rate continued to be significantly associated with a lower obesity rate (OR = 0.28; 95 % CI: 0.08, 0.98). Therefore, the difference between New England and other regions may be related to regional differences in per capita income, which cease to be correlated with women’s obesity when New England cases are excluded from the model. Additionally, regional differences in infant mortality rate during the Great Depression outside of New England may have influenced women’s odds of obesity in later life.

**Table 4:** Regional factors associated with adult obesity: odds ratios from logistic models at age 50–61 among women

	Model 1		Model 2		Model 3		Model 4		Model 5		Model 6	
Birth region fixed effects	OR	95 % CI	OR	95 % CI	OR	95 % CI	OR	95 % CI	OR	95 % CI	OR	95 % CI
Yield of wheat / 10	1.12	0.83, 1.51									1.13	0.76, 1.69
Yield of corn / 10	1.10	0.96, 1.26									1.18	0.94, 1.48
Yield of hay / 10	0.15	0.00, 11.93									0.05	0.00, 8.85
Yield of oats / 10	1.01	0.81, 1.25									1.06	0.83, 1.34
Per capita income / 1000			1.83	1.04, 3.23					2.37	1.01, 5.52	0.91	0.21, 3.97
Manufacturing employment index					1.00	1.00, 1.00					1.00	1.00, 1.00
Nonmanufacturing employment index					1.01	1.00, 1.02					1.01	0.99, 1.02
Infant mortality rate / 100							0.27	0.08, 0.96	0.57	0.13, 2.43	1.02	0.13, 8.07

Abbreviations: *CI* confidence interval, *OR* odds ratio, *SD* standard deviation

All models control for individual characteristics, including birth date, race-ethnicity, marital status, education, income, net wealth, lifetime occupation, current residential place, smoking status, physical activity, mother’s education, early life financial status, father lost job, received financial help, and family moved for financial reasons

## Discussion

The study of early-life determinants of later-life health has taken off since Caldwell’s pioneering work [32], with recent work demonstrating the influence of early-life factors such as maternal education on children’s health and survival [33–37]. In a review of the literature on early-life predictors of obesity, Brisbois and colleagues identified three prenatal factors--maternal body mass index, maternal smoking, and maternal weight gain during pregnancy—that contribute to obesity in adulthood [8]. Prenatal factors may affect adult obesity “through programming of structural and functional abnormalities of various endocrine systems” [38] and programming the mass and distribution of adipose tissue [8]. Greater maternal education, in particular, may be correlated with better health literacy and health behaviours, greater receptivity to modern medical treatments, and a more sanitary environment [37, 39–41]. As more educated mothers have better health literacy and health behaviour, they should be better at managing risk factors for obesity during early childhood, such as early rapid growth and early adiposity rebound [8]. Indeed, recent data from Western birth cohorts confirm an association between higher maternal education and a more favorable growth trajectory among their children—higher weight at birth, but lower chances of overweight and obesity in childhood and adolescence [42, 43]. Moreover, the lower smoking prevalence among more highly educated mothers means that their children will be less likely to experience early rapid growth and early adiposity rebound, which are correlated with greater obesity risk in adulthood [8, 44].

Whereas prior research on maternal education exemplifies lasting effects of individual-level early-life characteristics on weight trajectories, we have hypothesized that regional differences also create lasting birth-region disparities in obesity risk that persist into mid-life and beyond, net of individual-level socioeconomic measures and health risk factors assessed at midlife. We have studied a cohort of Americans born during the Great Depression, when the country experienced strong economic upheaval and economic development and productivity varied greatly across regions. Regional variation in these and other factors may have produced regional disparities in the prevalence of obesity that persisted into adulthood. Consistent with this argument, we have found that women who were born in the Mountain division were least likely to be obese in both middle and late adulthood, even after adjusting for mid-life sociodemographic characteristics and health behaviors. During the Great Depression (*i.e.*, at the time this cohort was born), the Mountain division was distinguished by low per capita income and high infant mortality as compared to other regions. In turn, low per capita income and high infant mortality in a woman’s birth region were correlated with lower odds of obesity in late life. Thus, regional measures of economic development may explain the regional differences we have detected in the odds of obesity among women in this cohort.

Why does low regional economic development (as indicated by low per capita income and high infant mortality) correlate with low odds of obesity? Women born in such regions may have been less likely to experience postnatal catch-up growth, and thus less likely to be obese in adulthood. The role of infant mortality rate is more complex. On the one hand, it may be an indicator of socioeconomic development and living standards, with effects similar to per capita income. On the other hand, high infant mortality may also suggest a mortality selection effect: if obesity (or the predisposition to obesity) is positively associated with mortality, then stronger mortality selection in regions with high infant mortality implies that surviving women will be disproportionately unlikely to be obese. Whatever the underlying mechanism, our analysis shows that differences in women’s obesity risk attributable to birth region persist into late life and are not explained by mid-life accumulation of socioeconomic or behavioral risk factors for obesity. Specifically, we find that women born in the Mountain division had the lowest odds of obesity even when obesity was assessed at age 66–77 and when the model was adjusted for childhood socioeconomic background as well as economic, demographic, and health covariates measured at ages 50–61. This finding is interesting and important because it suggests a persistent influence of birth region factors on adult health status, beyond any similar influences of early-life individual characteristics.

The effects of birth region and mother’s education are evident among women, but not among men. Furthermore, an analysis of the pooled sample confirms that the size of the maternal education effect differs significantly by gender. Both social and biological mechanisms may contribute to the gender disparity in the effect of early-life conditions. First, due to cumulative risks and disadvantages over the life course, women are more vulnerable to early-life disadvantages in family background [6, 23]. Second, due to social pressure for women to be slim, especially among women of higher socioeconomic status, women from advantaged backgrounds could be more likely to use healthy weight control methods (diet and exercise) to maintain their body size than women born in families with lower status [7]. Men, however, face less social pressure to be slim, regardless of social class. On the contrary, physical robustness is a sign of masculinity. Third, there may exist gender differences in “the body’s encoding and storage of early harmful exposures [23]”. For example, some studies found the Dutch famine affected women's but not men’s body mass index [23]. The existence of gender differences in biological response to early-childhood adversity is further substantiated by the significance of birth region effects, where both men and women experience the same characteristics of each birth region but experience different consequences in the case of late-life obesity.

To our knowledge, this is the first study to investigate gender differences in the effects of early life conditions, comprising both regional and individual or family characteristics, on obesity risk in late middle age and late life. Limitations of our analysis suggest promising new directions for research on regional variation in obesity risk. First, the HRS data we have used include respondents born during or immediately after the Great Depression, but did not include a comparable cohort of respondents born before the Great Depression that could have been included in an analysis of the Great Depression’s effects. Future research should explore whether this or other economic shocks shape regional variation in obesity within birth cohorts. Second, our operationalization of the birth region was necessarily limited to nine Census divisions, a sample size that precluded multilevel modeling due to the risk of biased estimation of region-level standard errors [45]. Future work should consider whether more finely-defined places of birth—such as states, counties, or cities—may have similar implications for obesity in later life when analyzed in a multilevel modeling framework. Third, we have not explored all possible sources of regional variation in later-life obesity risk, such as racial composition of the birth region, availability of resources supporting maternal and child health, or exposure to harmful pollutants. Fourth, we have relied on self-reported measures of height and weight, meaning that reporting errors influenced the calculation of BMI, and that unobserved differences in height and weight misreporting across birth region may have contributed to the regional differences in BMI we have found. Finally, our analysis of early-life effects used data collected retrospectively at ages 50 and older, meaning that a sizeable share of respondents were excluded due to missing values on retrospective measures of early-life socioeconomic status, and that the implications of birth region characteristics for physiological development and socioeconomic advancement in early life could not be observed. Further studies are needed to collect prospective data on children and young adults’ sociodemographic circumstances, track birth region variation in the body weight among such a cohort, and assess if this variation is mediated by social and biological pathways emerging before midlife.

## Conclusions

Early life conditions may influence the likelihood of obesity in early and middle adulthood [3–5]. The long-lasting effect of early-life conditions, however, may differ by gender and life course stage. We find that birth region and mother’s educational attainment shape obesity risk among women, but not men; and that the effects of these characteristics persist among women from late-middle age (ages 50–61) to late life (ages 66–77). These findings suggest that improving mothers’ education and controlling for risk factors tied to region, rather than to individuals or families, may have long-term benefits for women’s health, as exemplified by obesity risk in this paper. For men, adult health behavior and risk factors may be more pertinent causes of late-life obesity than early-life conditions.

## Additional file

Additional file 1: Tables S1-S4. Additional tables of regression results.

## Abbreviations

BMI: Body mass index
CI: Confidence interval
HRS: Health and retirement study
OR: Odds ratio
SD: Standard deviation
